# Shi's Yi-Qi Bu-Shen Tong-Luo decoction combined with manipulation in the management of wrist stiffness after distal radius fracture: Study protocol clinical trial (SPIRIT Compliant)

**DOI:** 10.1097/MD.0000000000019308

**Published:** 2020-02-28

**Authors:** Hai-Yang Wu, Yi-Ru Wang, Jing Gui, Guo-Wei Wen, Zhen-Yin Tang, Ping Liu, Jun-Hao Wu

**Affiliations:** a Huangpu Branch, Shanghai Ninth People's Hospital Affiliated to Shanghai Jiaotong University; b Longhua Hospital Affiliated to Shanghai University of Traditional Chinese Medicine, Shanghai, China.

**Keywords:** distal radius fracture, manipulation therapy, Shi's Yi-Qi Bu-Shen Tong-Luo decoction, traditional Chinese medicine, wrist stiffness

## Abstract

**Introduction:**

Wrist stiffness is a common sequela of distal radial fractures. Manipulation is generally used and effective, but problems exist, such as intense pain, swelling during the process of manipulation and long treatment period. Therefore, a combinative therapeutic strategy is necessary to benefit rehabilitation after distal radius fracture. Shi's Yi-Qi Bu-Shen Tong-Luo decoction (BTD) combined with wrist manipulation has been used in the Shanghai Ninth People's Hospital Huangpu Branch in last few decades. BTD has potential therapeutic effects on rehabilitation after distal radius fracture, which should be evaluated by rigorous clinical trial.

**Methods/design:**

A randomized, double-blind, placebo-controlled clinical trial will be conducted to determine the efficiency of BTD in relief of wrist stiffness and pain and function rehabilitation. A total of 80 wrist stiffness patients with or without pain and edema will be enrolled, and treated with wrist manipulation plus BTD or placebo for 4 weeks. The primary outcome measure is the Cooney wrist score. The second outcome measures include pain numerical rating scale, patient rated wrist evaluation, 36-item short form health survey questionnaire, and side effects.

**Discussion:**

Although BTD has shown effects on rehabilitation after distal radius fracture in the Shanghai Ninth People's Hospital Huangpu Branch for decades, the universality of this efficacy needs evaluated. The results of this trial will provide a convincing evidence.

**Trial registration:**

ChiCTR2000029260, January 19, 2020.

## 1 Introduction

Distal radius fracture (DRF) is an increasingly common upper limb injury. A certain percentage of complications occur frequently after fracture healing regardless of surgical or conservative treatment. Common complications include wrist instability, stiffness, nonunion, tendon irritation, infection, and local pain syndrome.^[1,2]^ Wrist stiffness is one of the most common and difficult sequela to treat, up to 30% of DRF patients.^[3]^ Manipulation is an effective treatment method, but there are problems such as intense pain, swelling and long treatment periods.^[4]^ Therefore, a combinative therapeutic strategy that could relieve pain, swelling and joint adhesion is necessary to benefit DRF patients after surgeries.

In recent years, traditional Chinese medicine (TCM) has played an important role in the rehabilitation of various kinds of fractures and arthritis. Research suggests TCM has analgesic and anti-inflammatory effects in patients with osteoarthritis.^[5]^ TCM also could relieve pain and swelling after fractures ^[6]^ and promote fracture healing in multi-regulatory pathways, such as Wnt/β-catenin, bone morphogenetic protein/Smad and mitogen-activated protein kinase pathway.^[7]^

Shi's Yi-Qi Bu-Shen Tong-Luo decoction (BTD) is a century-old historical TCM prescription, including Astragali Radix, Radix Angelicae Sinensis, Flos Carthami, Rhizoma Chuanxiong, Taxilli Herba, Eucommiae Cortex, Epimedii Folium, Olibanum, Stephaniae Tetrandrae Radix (details in Table 1). It has been used to improve joint pain and swelling in the DRF late stage in clinical treatment for decades. According to the TCM theory, BTD could supplement Qi and activate blood circulation, then promote release of adhesion and recovery of joint function.^[8,9]^ Unfortunately, its efficacy remains unverified with rigorous research and exact data.

**Table 1:**
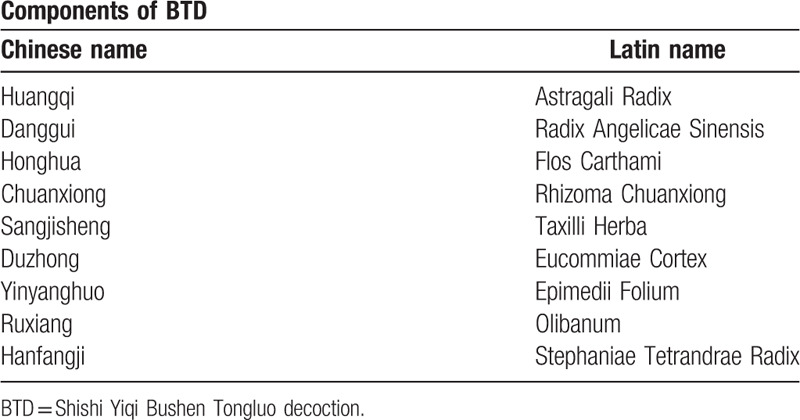
Components of BTD (a separate Word file).

Based on this, we propose a randomized, double-blind, placebo-controlled trial to estimate the efficiency and security in managing wrist stiffness of BTD on DRF patients’ rehabilitation. This protocol finished in January 5, 2020 (Version 1).

## 2 Methods/design

### 2.1 Study design

A prospective single-center, double-blinded, placebo-controlled, randomized clinical trial with 2 parallel arms. The determination of this study is to evaluate the effectiveness and safety of BTD in managing wrist stiffness after DRF. Manipulation by specific therapist is the preliminary treatment, will be used in both groups. In this study, we will recruit 80 wrist stiffness patients due to DRF from Huangpu Branch, Shanghai Ninth People's Hospital affiliated to Shanghai Jiaotong University.

All participants will be randomized into BTD group or placebo group equal in patient number. Patients in BTD group will receive both BTD (200 mL once, twice a day) and manipulation (30 minutes once, twice a week), while patients in placebo group will receive BTD placebo (200 mL once, twice a day) and manipulation (30 minutes once, twice a week) for continuous 4 weeks. All patients were treated by the same therapist in the entire manipulation.

### 2.2 Ethical issues

The care provider will invite the patient to participate the trial, tell them in detail why we should take this trial and what kind of rights, obligations and risks they will have if they participate the trials. And the care provider will give them a written informed consent. Only the patients fully understand and sign the informed consent, can they participant the trial. The trial will be conducted in accordance with the Declaration of Helsinki and Ethical Guidelines for Clinical Research, and the trial protocol has been approved by the Research Ethical Committee of Huangpu Branch, Shanghai Ninth People's Hospital affiliated to Shanghai Jiaotong University (approval number: 2019-Q-026). And the protocol has been listed on Chinese Clinical Trial Registry (ChiCTR2000029260).

### 2.3 Study participants

DRF participants from the Huangpu Branch, Shanghai Ninth People's Hospital affiliated to Shanghai Jiaotong University will be recruited. Recruitment poster and website advertisements will be publicized. The planned recruitment period is 12 months.

### 2.4 Criteria

#### 2.4.1 Inclusion criteria

Participants meeting the following criteria will be included.

(1) patients with diagnosis of DRF and age from ≥18 and ≤75
(2) receive conservative treatment (such as plaster external fixation, small splint fixation and external fixation stent) or surgery
(3) over 6 weeks after the fractures at least, X-ray shows blurred fracture line and well callus growth, external fixation such as plaster and support is removed
(4) wrist stiffness, with or without swelling, pain, range of joint activities is significantly lower than the healthy side
(5) able to understand and sign the informed consent and ensure compliance

#### 2.4.2 Exclusion criteria

(1) fracture is unstable
(2) with nerve injury
(3) abnormal liver and kidney function
(4) pregnancy, or have no plans of pregnancy, or feeding women
(5) severe chronic or acute disease
(6) addictive to alcohol

### 2.5 Interventions

#### 2.5.1 TCM intervention

In BTD group, patients will be instructed take the 200 mL BTD liquid medicine orally twice a day for 4 weeks. While patients in the placebo group will take BTD placebo in the same way.

The BTD liquid medicine will be manufactured, packaged and labeled by TCM pharmacy of Huangpu Branch, Shanghai Ninth People's Hospital affiliated to Shanghai Jiaotong University with automatic Chinese medicine decocting machine. The crude herbs include Astragali Radix (Huangqi, 30 g), Radix Angelicae Sinensis (Danggui, 12 g), Rhizoma Chuanxiong (Chuanxiong, 9 g), Taxilli Herba (Sangjisheng, 12 g), Flos Carthami (Honghua, 15 g), Eucommiae Cortex (Duzhong, 12 g), Epimedii Folium (Yinyanghuo,12 g), Olibanum (Ruxiang, 9 g), Stephaniae Tetrandrae Radix (Fangji, 15 g). Process as follows:

(1) Decoction: every 14 prescriptions of BTD are packed together. All the herbs mentioned above are put into automatic Chinese medicine decocting machine for 1 hour. Herbal dregs are filtered out, and herbal filtrate collected.
(2) Packing: Herbal filtrate is divided into 28 plastic bags, 200 mL liquid in each bag
(3) Directions: take orally 1 bag at 1 time and twice a day.

The BTD placebo contains 5% BTD and 95% bitterant, lactose edible essence, pigment (such as lemon yellow, caramel pigment, and sunset yellow) and starch, and exhibit shape, smell, color and taste similar to those of BTDs. Bag the placebo as the BTD packing method.

### 2.6 Manipulation intervention

At the same time, patients in both 2 groups will receive hand manipulation (30 minutes, twice a week) by the same therapist. Specific steps are as follows (take the right stiffness hand of seated patient as an example):

(1) The therapist holds the lower part of the forearm with his left hand and the distal palm with right hand, pulls the wrist bone to the distal continuously, against the traction from light to stronger until there is obvious resistance;
(2) The therapist fixes the forearm and slide the proximal palm to the palmar, and requires the patient to straighten actively the wrist at the same time;
(3) The therapist slides the proximal palm to the dorsal, and requires the patient to flexes actively the wrist at the same time;
(4) The therapist slides to the ulnar side, requires the patient to bend wrist radially at same time;
(5) The therapist slides to the radial side, requires the patient to bend wrist to ulnar direction at same time. Rotate the forearm while the patient actively against resistance. Each step lasts for about 20 seconds, and no obvious pains in the whole progress should be ensured.

### 2.7 Forbidden treatments and drugs

(1) any other TCM methods (herbs except for BTD or BTD placebo, acupuncture, cupping, plaster, and etc) are not allowed
(2) once patients are in the study period, DRF surgery is not allowed

### 2.8 Visiting plan

At baseline (Visit 1), patients begin to receive manipulation plus BTD or BTD placebo and laboratory tests. Patients will visit again at week 2 and 4 (Visit 2 and 3) and perform laboratory tests at week 4. Both Medicine and manipulation intervention will be finished at week 4. Then the follow-up visit (Visit 4) will be taken at week 12. Every patient should visit and do laboratory tests within 3 days after the original time point (details in Table 2). During the study period, all the treatments and drugs patients have used should record in details in CRFs.

**Table 2:**
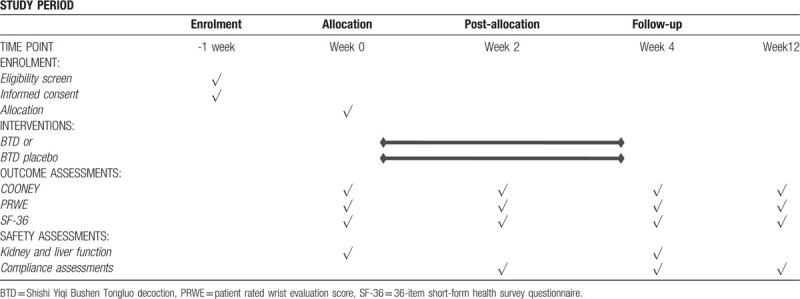
Schedule of enrollment and assessments (a separate word file).

### 2.9 Randomization and allocation

The random number list will be generated through Statistical Packages of Social Sciences (SPSS) software (version 24.0) in a 1:1 ratio by a trained statistician. The list contains both the sequence number of inception and the group of BTD or BTD placebo. This statistician will contact and provide the randomization list for medicine pharmacy of Huangpu Branch, Shanghai Ninth People's Hospital affiliated to Shanghai Jiaotong University. Medicine pharmacy will produce the medicinal liquid and paste the sequence number label on plastic bags of BTD or placebo as the list.

The statistician and pharmacy will preserve the randomization list to maintain concealment. And the other investigators will be unable to know the sequence numbers and group of each participate until the trial completed. Medicine with labels will be stored in a locked and individual room. A trained administrator will charge the medicine and the key.

When a participant will be recruited in, the investigator will make a central telephone call and give him/her the medicine (BTD or the placebo) from the administrator.

### 2.10 Blinding

The stuff in the medicine pharmacy only provide drugs, and never participate in the other parts of this study. The investigators, clinicians, therapist, statisticians (analyze outcomes), and participants are unconscious of the group assignment information until the entire trial completed.

### 2.11 Adverse event

When an adverse event is claimed, we will record it and provide an appropriate treatment. Clinicians will provide emergency services in case of serious adverse events, and report it to the Institutional Review Board within 24 hours. According the condition of adverse event, the blinding might be broken by the co-decision of main trial investigator and leader of ethic committee. Intervention information will be offered by statistician.

### 2.12 Outcomes

#### 2.12.1 Primary outcome

##### 2.12.1.1 Cooney wrist score (from baseline to week 4)

The Cooney wrist scoring system used for the assessment and functional evaluation of the wrist. It includes 4 parts, pain symptoms (25 points), functional status (25 points), grip strength (25 points), range of motion (25 points). The total score of Cooney is 100 points. The higher Cooney score, the better recovery of wrist function. The evaluation criteria is: excellent, 90 to 100 points; good, 80 to 89 points; fair, 65 to 79 points; poor, 0 to 64 points (details in Table 3).

**Table 3:**
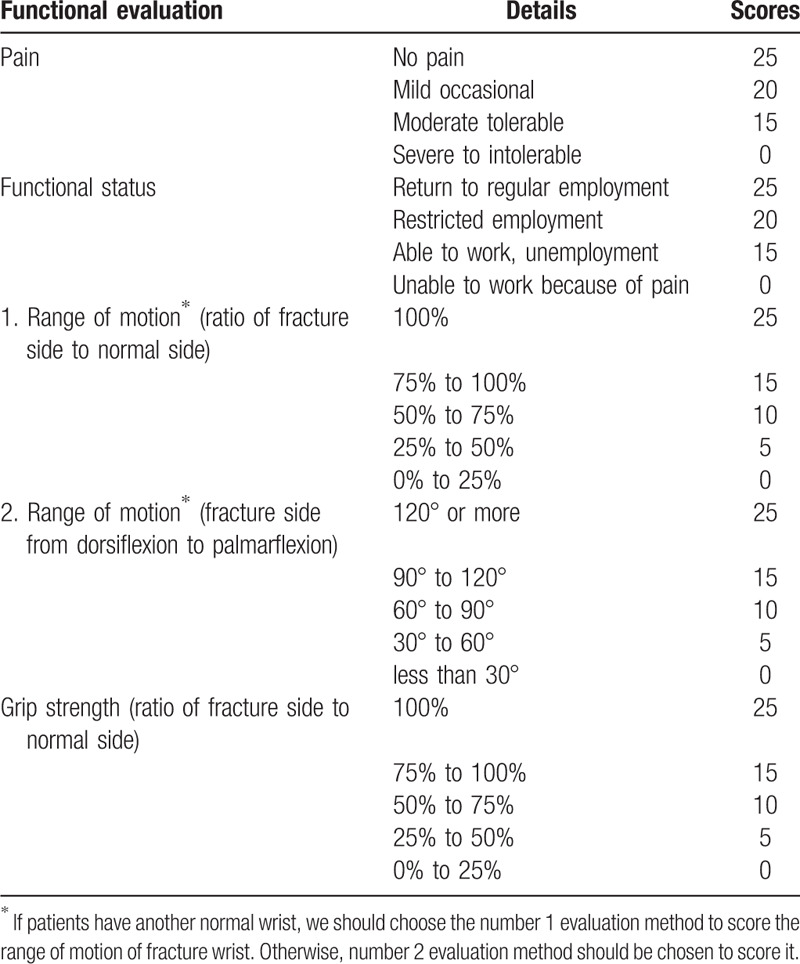
Cooney wrist score (a separate word file).

#### 2.12.2 Secondary outcomes

##### 2.12.2.1 Cooney wrist score (from baseline to week 2 and 12)

Cooney wrist score from baseline to week 2 and 12 will be calculated as one of the secondary outcomes.

##### 2.12.2.2 Patient rated wrist evaluation (PRWE) score (from baseline to week 2, 4, and 12)

The PRWE score questionnaire is designed specifically for evaluation of distal radial fractures that rates wrist function sensitively using a range of questions in 2 parts concerning the patient's pain and function. The PRWE score included 15 items: 5 items related to pain (4 related to the intensity of pain, 1 related to the frequency of pain), 6 items related to special activities, and 4 items related to daily activities which can reflect difficulty in self-care, household activities, work and recreation. Each small item can be marked with a score. The total score is 100, which is calculated by dividing the sum of 10 items scores related to activity and function by 2 (out of 50), plus the total score for the pain subcategory, to give a score on a scale of 0 to 100. The higher score, the more severe pain and dysfunction. We enquired patients to fill out the questionnaire based on their wrist pain, special activities and difficulties in daily activities in the past week, and then the specific score was calculated by the doctor to get the final score. The PRWE is the frequently outcome measure available for DRFs.

##### 2.12.2.3 36-Item short form health survey questionnaire (SF-36) (from baseline to week 2, 4, and 12)

SF-36 is an implement for assessing physical status of patients which consists of 36 items in 8 types, including role physical, physical function, bodily pain, vitality, and global health, social function, mental health and role emotional.

### 2.13 Safety assessments

To assure the safety of participants, the adverse effects will be reported and analyzed. At baseline and after BTD intervention, patients’ kidney and liver function will be tested. Additionally, side effects of TCM herbs will be record along the trial, such as gastrointestinal reaction, abdominal pain, dizzy, rash, and and so on. Participants were free to withdraw from the study at any time. The data collection process will be administered by the project director.

### 2.14 Participant timeline

**Figure 1:**
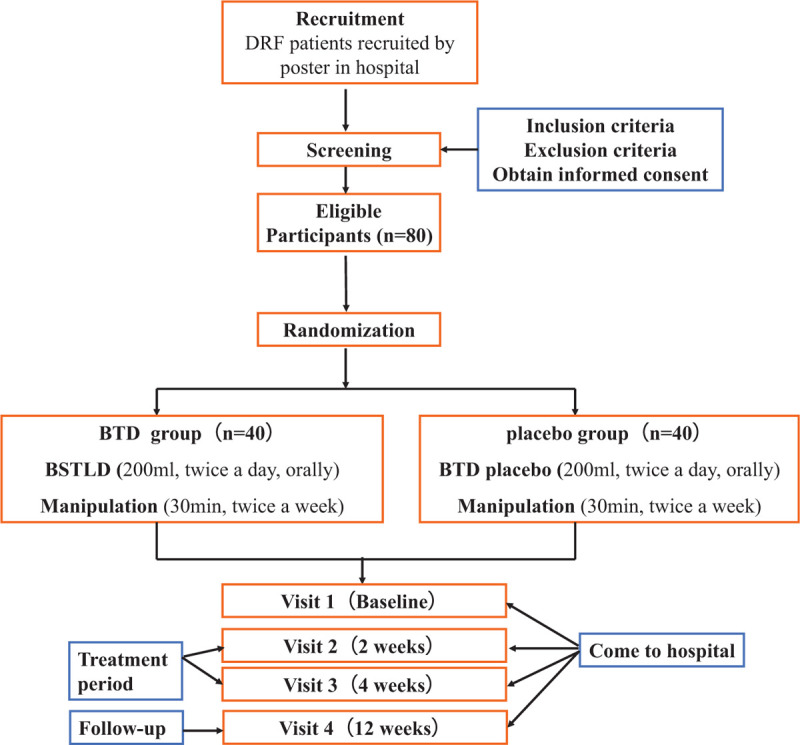
Project overview. BTD = Shi's Yi-Qi Bu-Shen Tong-Luo decoction, DRF = distal radius fracture.

Recruitment will start in March 2020 and end in March 2021. Last visit will be finished before June 30, 2021. The recruitment process is shown in Figure 1, and the schedule is shown in Table 1.

### 2.15 Data collection and monitoring

All participants will receive treatment of manipulation plus BTD or BTD placebo for 4 weeks and followed-up for another 8 weeks. Two statisticians independently will record 4 rounds of disease assessments (the baseline, week 2, week 4, and week 12) and 2 rounds of safety assessments (the baseline and week 4) in Epidata software (Version 3.1). Disagreement will be solved by discussion with a third statistician.

### 2.16 Quality control

Huangpu Branch, Shanghai Ninth People's Hospital affiliated to Shanghai Jiaotong University control the quality of this whole search. Training will be conducted to study the process of the entire trial before the trial. All the physicians, therapist and evaluators will receive this training together. A specific investigator will check the CRFs weekly to make sure the study quality.

### 2.17 Sample size calculation

Sample size was calculated according to the Cooney wrist score, based on previous studies (Zhang et al 2014^[10]^). 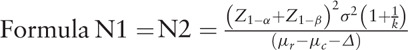 was used, N1 and N2 mean the patient numbers in BTD group and placebo group; 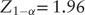 when *α* = 0.05; 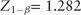 when 1-*β* = 0.90; *σ* (standard deviation of 2 groups) = 7.7; *k* = 1; 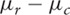 (the difference of Cooney wrist score in BTD and placebo group) = 4; Δ = 0. Therefore, we decided to include 80 patients in the study (40 of them in each group).

### 2.18 Statistical analysis

All data analyses will be accomplished using SPSS software (version 24.0). Continuous data are presented as mean ± standard deviation and categorical data as frequencies. Continuous variables following the normal distribution will be analyzed by Student *t* test; otherwise, nonparametric tests will be used to compare group differences. Statistical test is 2-sided and *P*-value <.05 will be defined as statistically significant. We will adopt multiple imputation to handle missing data statistically. A subgroup analyses will be conducted when necessary. Intention-to-treat approach and last-observation-carried-forward method will be applied to deal with the missing values.

## 3 Discussion

TCM has shown effective in treating fractures and injures.^[11]^ BTD consists of Astragali Radix, Radix Angelicae Sinensis, Flos Carthami, Rhizoma Chuanxiong, Taxilli Herba, Eucommiae Cortex, Epimedii Folium, Olibanum, and Stephaniae Tetrandrae Radix. BTD has been used to treat delayed union of fractures and degenerative bone diseases, such as cervical and lumbar disc degeneration, osteoporosis, osteopenia and osteoarthrosis.^[12,13]^ Experimental studies^[14]^ have found that the TCM formula similar to BTD could accelerate the recovery process after fractures, also could reduce inflammation and swelling of joints on inflammatory arthritis model mice.^[15]^ Additionally, experimental study has shown that astragalin, 1 composite of BTD, could attenuate synovial inflammation via MAPKs and AP-1 pathways.^[16]^

We use the BTD to treat DRF patients in clinical strategy and this formula works. However, we reviewed the major databases and found no literature to prove that BTD could be benefit to the rehibition of wrist stiffness after DRF.

Based on this, we designed this double-blind randomized controlled trial to explore the effects and safety of BTD. Cooney wrist score is used to evaluate the improvement in pain and wrist motion before and after treatment. The PRWE score measures changes in pain, function and daily life of both physicians’ objective assessments and patients’ subjective feelings. The SF-36 score is used to assess the physical and mental health of patients, and the safety of BTD is also monitored along the study.

This research will answer whether BTD combined with manipulation is an effective strategy in management of wrist stiffness after DRF. Its positive results will offer patients better functional recovery. Furthermore, clinical physician will have an optional treating strategy.

## Author contribution

HYW conduct the trial and draft the manuscript. JHW and PL supervised and coordinated the clinical trial. HYW, GWW, and JG are responsible for recruiting the participants. ZYT is the specific manipulation therapist. All authors read and approved the final manuscript. HYW and YRW conceived of the study and revised the manuscript critically for important intellectual content.
